# Effect of increased body mass index (BMI) on time to tumour progression (TTP) in unresectable metastatic colorectal cancer (mCRC) patients treated with bevacizumab-based therapy

**DOI:** 10.1007/s12032-013-0679-4

**Published:** 2013-08-07

**Authors:** N. Faruk Aykan, Ibrahim Yildiz, Fatma Sen, Leyla Kilic, Serkan Keskin, Rumeysa Ciftci, Senem Karabulut, Burak Sakar, Rian Disci

**Affiliations:** 1 Department of Medical Oncology, Institute of Oncology, Istanbul University, Istanbul, Turkey; 2 Department of Biostatistics, Institute of Oncology, Istanbul University, Istanbul, Turkey

**Keywords:** Colorectal cancer, Body mass index, Bevacizumab, Time to progression

## Abstract

High BMI is a well-known risk factor for the development and recurrence of several solid tumours, including CRC. Obesity is associated with increased levels of vascular endothelial growth factor (VEGF). Bevacizumab is the main targeted therapy for inhibiting tumour angiogenesis by blocking the VEGF/VEGF receptor pathway. Elevated VEGF in obese patients might provoke resistance to anti-VEGF therapy. We evaluated the efficacy of bevacizumab on TTP among mCRC patients through stratifying them according to their BMI. Patients with mCRC who had been treated with fluoropyrimidine-based combination chemotherapy with bevacizumab were included in the study. Patients were assigned according to their BMI before initiation of therapy (group A: BMI < 25 kg/m^2^, group B: BMI ≥ 25 kg/m^2^). Multivariate analysis was performed to evaluate the risk of tumour progression. Between April 2007 and June 2011, 80 patients were treated with chemotherapy and bevacizumab as first-line therapy (*n* = 37 for group A, *n* = 43 for group B). Tumours in 56.3 % of the patients in group A (*n* = 21) and 76.3 % of the patients in group B (*n* = 33) progressed during a median 10-months (3–57 months) follow-up. The median TTP was 11.7 months in the group A and 6 months in the group B (*p* = 0.004). In a multivariate analysis, high BMI (≥25 kg/m^2^) was associated with significantly shorter TTP (*p* = 0.01; HR: 4.37). High BMI among mCRC patients treated with bevacizumab is associated with shorter TTP. Further study in larger databases is warranted for confirming the negative prognostic effect of obesity during treatment with anti-VEGF agents.

## Introduction

In recent decades, the prevalence of obesity has increased worldwide in all age groups [1]. Increasing epidemiological evidence has demonstrated that obesity is associated with an increased risk of cancer, especially colon cancer [2–4]. Several factors, such as insulin resistance, increased levels of leptin, plasminogen activator inhibitor-1, endogenous sex steroids, decreased levels of adiponectin, and chronic inflammation, are involved in carcinogenesis and cancer progression [5].

However, a few studies that have investigated the influence of BMI on the outcomes of colon cancer patients have reported inconsistent findings [6–11]. Despite support for the importance of obesity and metabolic syndrome as risk factors for the development of colorectal cancer, data are equivocal for the effects on colorectal cancer progression and outcome [6–8]. Several studies have reported decreased survival and increased recurrence in patients with insulin resistance or high BMI [7–9], whereas other studies have not [6, 10, 11]. Although high BMI was associated with better median OS in the CAIRO study, CAIRO2 study did not show this association [11]. Consequently, testing the role of BMI in patients receiving targeted therapy is an actual subject of research.

Bevacizumab is a humanised monoclonal antibody (mAb) against vascular endothelial growth factor (VEGF), which is the major mediator of angiogenesis. Activation of the VEGF/VEGF receptor axis triggers multiple signalling networks responsible for endothelial cell survival, mitogenesis, migration, and differentiation [12]. Elevated serum VEGF levels have been observed in overweight or obese patients [13, 14]. The BMI of patients with metastatic CRC at treatment initiation predicts treatment outcomes. Guiu et al. [15] reported that high visceral fat area (VFA) measured by computed tomography independently predicted poorer outcome in a retrospective series of patients given first-line bevacizumab-based therapy for metastatic colon cancer.

Although not all patients diagnosed with metastatic CRC benefit from anti-VEGF antibody treatment, there are currently no biomarkers available to predict the efficacy of anti-angiogenic therapies. VEGF plays an important role as an endothelial mitogen in tumour angiogenesis, and increased levels of VEGF may contribute to poorer outcomes in cancer in obese subjects [16]. Thus, BMI may be a prognostic factor for poor outcomes in metastatic CRC patients receiving bevacizumab-based treatment. On the other hand, standard bevacizumab dose may be questionable for obese patients. We performed a study of the associations between BMI and clinical outcomes in consecutive patients administered bevacizumab-based treatments for metastatic CRC.

## Patients and methods

### Eligible patients

From April 2007 to June 2011, 80 consecutive patients with metastatic colorectal adenocarcinoma treated with fluoropyrimidine-based combination chemotherapy plus bevacizumab at the Institute of Oncology, Istanbul University (Istanbul, Turkey), were included in the study. Patients, age between 18 and 80 years old with histologically confirmed locally advanced or metastatic CRC, were determined. Those with one or more unidimensionally measurable lesions, who were not amenable to curative resection, with an Eastern Cooperative Oncology Group (ECOG) performance status of 0–2, no prior history of cancer (except basal cell carcinoma or carcinoma in situ of the cervix), adequate bone marrow (white absolute neutrophil count, ≥1,500/mm^3^, platelet count ≥100,000/mm^3^) and liver function (total bilirubin ≤1.5 mg/dl and alanine and aspartate transaminase levels <5 X upper limit of normal), and a life expectancy of longer than 3 months were included. Patients who received bevacizumab as a second-line treatment were also included. Patients received one of the following treatment regimens: simplified LV5FU2 (leucovorin 400 mg/m^2^, followed by 5-fluorouracil as a 400 mg/m^2^ bolus and a 2,400 mg/m^2^ infusion over 46 h), modified FOLFOX regimen (simplified LV5FU2 regimen plus oxaliplatin 85 mg/m^2^), FOLFIRI (simplified LV5FU2 regimen plus irinotecan 180 mg/m^2^ every 2 weeks), XELOX (capecitabine 1,000 mg/m^2^, b.i.d., p.o., days 1–14 plus oxaliplatin 130 mg/m^2^ every 3 weeks), or XELIRI (capecitabine 1,000 mg/m^2^, b.i.d., p.o., days 1–14 plus irinotecan 240 mg/m^2^ every 3 weeks). Bevacizumab was administered at a dosage of 5 mg/kg every 2 weeks or 7.5 mg/kg every 3 weeks. The dosages of chemotherapeutic agents were determined according to the measured body surface area (BSA); however, for patients with BSA over 2 m^2^, dosages were adjusted according to 2 m^2^. For bevacizumab, no dosing reduction was accomplished, i.e. patients received the exact planned dose for their actual weight. All of the patients had unresectable liver metastases associated with primary tumours at the initial consultation. Patients who underwent hepatic resection and local ablative therapies, chemo-embolisation, or radio-embolisation were excluded. Pregnant or breast-feeding women were excluded. Other key exclusion criteria were as follows: clinically significant cardiovascular disease; exclusive bone metastasis; use of full-dose anti-coagulants or thrombolytics; known CNS metastases; serious non-healing wound, ulcer, clinically significant bleeding diathesis, or coagulopathy.

Tumour response was evaluated using a computed tomography scan or magnetic resonance imaging depending on which imaging methods were used at baseline. Tumour responses and progression were determined every 8 weeks using the Response Evaluation Criteria in Solid Tumours [17] by the investigators and classified as follows: complete response (CR), partial response (PR), stable disease (SD), or progressive disease (PD). The tumour response after 2 months of chemotherapy was used for statistical analysis. Patients with either CR or PR were classified as responders, and patients with SD or PD were considered non-responders. This retrospective study was approved by our institutional review board.

Baseline demographic, clinical, and laboratory data, including age, gender, performance status, tumour marker levels, K-ras mutation status, and treatment details, were collected retrospectively for all patients using uniform database templates to ensure consistent data collection.

### Statistical analyses

SPSS version 16.0 (SPSS Inc., Chicago, IL) was used for the statistical analyses. The follow-up duration was calculated from the date of the first bevacizumab administration to the date of death or last follow-up visit. The time to progression (TTP) was calculated as the period from the initiation of treatment to the first observation of disease progression or to disease-specific death. The influence of putative prognostic factors on the progression of disease was evaluated by univariate analysis. Fifty-four (67.5 %) of the 80 patients with complete clinical and pathological data experienced disease progression during follow-up. TTP was evaluated with Mann–Whitney U test. Descriptive, univariate, and multivariate analyses were performed for those 54 patients with progression. Univariate Cox proportional-hazards models of all the potential baseline predictors were built to compute the hazard ratios (HR) with 95 % confidence intervals (CI). A multivariable binary logistic regression analysis was accomplished using a stepwise algorithm including age (years; <60 vs. ≥60), BMI (<25 vs. ≥25), primary tumour status (intact versus non-intact), type of chemotherapy regimens (irinotecan based versus oxaliplatin based), K-ras mutational status (wild type versus mutated), carcinoembryonic antigen (CEA) level (median, <28 vs. ≥28), and number of disease sites. For BMI, patients were categorised as underweight/normal weight (BMI < 25 kg/m^2^), or overweight/obese (BMI ≥ 25 kg/m^2^). All P values were two-sided.

## Results

At the time of the final follow-up, 4 (5 %) within 54 patients (67.5 %) who exhibited tumour progression were deceased due to disease-related factors. Remaining 26 (32.5 %) patients had no evidence of progression during the last evaluation.

The clinical and demographic data of 54 metastatic CRC patients who had disease progression during chemotherapy with bevacizumab were summarised in Table 1 and 2. The median age for these patients was 60.5 years (range 34–78); 59.3 % (*n* = 32) of the patients were male, and 40.7 % (*n* = 22) were female. Distant metastasis was present in 63 % of the patients at the time of diagnosis. Adjuvant chemotherapy was administered in 20 (37.0 %) patients. The primary location of the malignancy was sigmoid colon and recto-sigmoid junction in 18 patients (33.4 %), rectum in 17 patients (31.5 %), right colon-caecum in 14 patients (25.8 %), left colon in 3 patients (5.6 %), and transverse colon in two patients (3.7 %). Twenty-nine (53.7 %) patients had K-ras mutations, and 25 (46.3 %) patients had no mutations.

**Table 1:** Basal characteristics of metastatic CRC patients who progressed during bevacizumab-containing chemotherapy

Characteristic	Patient number (%)	Characteristic	
*BMI*		*Age*	
BMI < 25	21 (38.9)	Median (years)	60.5
BMI ≥ 25	33 (61.1)	(range)	(34–78)
*Sex*		*CEA*	
Male	32 (59.3)	Median (ng/ml)	5
Female	22 (40.7)	(range)	(0.8–20,300)
*KRAS analysis*		*CA 19–9*	
Mutant	29 (53.7)	Median (ng/ml)	39.5
Wild	25 (46.3)	(range)	(1–15,100)
*Number of metastatic site*			
1	33 (61.1)		
>1	21 (38.9)		

*BMI* body mass index, *CEA* carcinoembryonic antigen, *CA 19–9* carbohydrate antigen 19–9

**Table 2:** Treatment details of patients with disease progression

Characteristic	*n* = 54 (100 %)
*Primary tumour resection*	
Yes	45 (83.3 %)
No	9 (16.7 %)
*No. of cycles*	
Median	10
(range)	(2–32)
*Combined drug*	
Oxaliplatin	19 (35.2 %)
Irinotecan	35 (64.8 %)
*Response to bevacizumab*-*based therapy*	
CR + PR	23 (42.6 %)
SD + PD	31 (57.4 %)

*CR* complete response, *PR* partial response, *SD* stable disease, *PD* progressive disease

All patients received bevacizumab-containing chemotherapy regimens as first-line therapy. The median number of treatment cycles was 10 (range 2–32). Oxaliplatin-based chemotherapy was administered to 19 patients (35.2 %), and irinotecan-based chemotherapy was administered to 35 patients (64.8 %) in combination with bevacizumab.

Height and weight were recorded before initiation of bevacizumab and used to assign patients to group A (BMI < 25 kg/m^2^) and group B (BMI ≥ 25 kg/m^2^). Baseline characteristics (age, gender, number of metastatic locations, CEA, and CA 19–9 levels and K-ras mutation status) and treatment details such as number of cycles, resection status of the primary tumour, type of chemotherapeutic agent combined with bevacizumab, and response to treatment of all patients are represented in Tables 1 and 2. Twenty-one (56.3 %) of 37 patients in group A and 33 (76.7 %) of 43 patients in group B progressed during a median of 10-months follow-up (range 3–57 months).

For 54 patients who had disease progression and complete clinical data concerning the variables included in univariate analysis, the influence of the 7 variables on TTP was evaluated with Mann–Whitney U test. Finally through grouping TTP according to the median value, the effect of these variables on TTP was analysed with binary logistic regression model. In univariate analysis, BMI ≥ 25 (*p* = 0.004) and higher number of metastatic sites (*p* = 0.032) were associated with poor TTP. The median TTP was 11.7 months in the group A and 6 months in the group B (*p* = 0.004).

A multivariate logistic regression model for TTP was constructed that included age, BMI, serum CEA level, K-ras mutational status, primary tumour status (intact or non-intact), chemotherapy regimens, and number of disease sites. The results are displayed in Table 3. The only independent prognostic factor for TTP was BMI (*p* = 0.01; OR, 4.37; 95 % CI, 1.34–14.78) for patients with metastatic CRC treated with bevacizumab.

**Table 3:** Logistic regression model of clinical features for predicting time to tumour progression (TTP)

Parameter	*n*	TTP (months) Median	Min.	Max.	Univariate analysis* *p* value
BMI, kg/m^2^					0.004
<25	21	11.70	5.68	19.02	
≥25	33	6.00	1.38	17.74	
CEA median (ng/ml)					0.566
<28	34	9.49	1.38	18.60	
≥28	20	7.26	3.68	19.02	
Age (years)					0.264
<60	27	9.49	3.25	19.02	
≥60	27	7.00	1.38	17.74	
Primary tumour					0.618
Intact	9	10.28	1.38	18.60	
Non-intact	45	7.52	2.00	19.02	
K-ras mutation status					0.683
Wild type	25	9.49	1.38	17.74	
Mutated	29	7.00	3.25	19.02	
Chemotherapy					0.683
Irinotecan-based	35	8.71	3.25	19.02	
Oxaliplatin-based	19	7.00	1.38	18.60	
No. of disease sites					0.032
<2	33	10.28	2.00	19.02	
≥2	21	5.72	1.38	17.74	

In multivariate analysis, the only independent prognostic factor for TTP was BMI (*p* = 0.01; HR 4.37; 95 % CI 1.34–14.78) for patients with mCRC treated with combination chemotherapy and bevacizumab

* Mann–Whitney U test

## Discussion

Recent phase III trials have shown that adding bevacizumab to a first-line conventional chemotherapeutic regimen improved progression-free survival and overall survival in patients with metastatic CRC [18, 19]. Despite extensive investigation, there are no validated predictive biomarkers of the efficacy of VEGF-targeted therapies, such as bevacizumab. Obese animal models have been shown to be resistant to anti-VEGF treatment [20], which suggests that increased amounts of visceral fat may be associated with high VEGF levels and resistance to bevacizumab-based regimens in patients with metastatic CRC [15].

In the present study, the median TTP was 11.7 months in the BMI < 25 group and 6 months in the BMI ≥ 25 group (*p* = 0.004). In addition, the multivariate analysis indicated that increased BMI was the most important predictor of progression in the patients receiving the bevacizumab treatment. Evidence accumulated over the past decade has clearly established excess body fat as a risk factor for colorectal cancer. Specifically, risk increases with increasing BMI in a sex-(greater risk for men) and site-specific (greater occurrence in the colon versus rectum) manner [21]. A number of plausible biological mechanisms are responsible for the observed associations, including increased insulin resistance, increased availability of insulin-like growth factor (IGF)-1 (which is mitogenic, proapoptotic, and proangiogenic and increases cell motility), and altered adipokine metabolism. These alterations result in increased leptin, which is mitogenic, anti-apoptotic, and proangiogenic, and decreased adiponectin, which is also anti-angiogenic and anti-inflammatory [5, 22].

Obesity is a well-established risk factor for developing CRC [23] and has been associated with increased mortality from colon cancer [24, 25]. Obesity is also associated with a sedentary lifestyle and typical Western diet, which are associated with increased rates of cancer recurrence and death among patients with a history of curative surgical resection for CRC [7, 26, 27]. The patho-physiology is not completely understood but may involve adipose tissue production of adipokines and proangiogenic cytokines, such as VEGF, which may promote cancer growth and dysregulated angiogenesis [13]. All of the studies evaluating the association between body mass and mortality caused by CRC have focused on patients at the time of diagnosis of localised diseases. To our knowledge, only one trial [15] has analysed the influence of obesity on the prognosis of metastatic CRC patients receiving VEGF-targeted therapy.

Obesity is strongly associated with changes in the physiological function of adipose tissue and may cause insulin resistance, chronic inflammation, and altered secretion of adipokines. Adipose tissue is now recognised to function as an endocrine and paracrine organ that releases cytokine-like polypeptides responsible for widespread biological effects [28, 29]. In particular, adipocytes produce insulin-like growth factor and multiple angiogenic factors, including VEGF and leptin [30]. Leptin exerts direct angiogenic effects [29, 30], upregulates VEGF mRNA expression [31], and induces VEGF-mediated angiogenesis by colonic epithelial cells [32]. Inflammatory cells infiltrating the adipose tissue and adipose stromal cells also contribute to VEGF production [30, 33]. Elevated serum VEGF levels have been reported in overweight and obese patients [13, 14].

Visceral fat accumulation has been shown to be associated with colorectal adenoma formation [34], colon carcinogenesis [35], and increased risk for developing colon cancer. The amount of visceral fat independently predicts disease-free survival in patients with resectable CRC [8]. The data strongly suggest that visceral fat may induce the accumulation of protumorigenic factors and be associated with poorer outcomes in patients with CRC.

The role of bevacizumab-based therapy (in combination with conventional chemotherapy) has been established as an option for metastatic CRC. Until now, no predictive biomarker has been available to direct treatment algorithms. Biomarkers are available for other mechanism-based therapies in metastatic CRC, including K-ras status and cetuximab therapy, in which measurement of the predictive biomarker clearly discriminates the subsequent treatment response. While using BMI as a ‘lifestyle biomarker’ may appear to be unconventional (compared with traditional gene or protein biomarkers), lifestyle is used as a predictive factor for treatment response of other cancer types; smoking status is used for directing anti-epidermal growth factor receptor (EGFR) therapy (erlotinib) in non-small cell lung cancer.

The limitations of our study include a relatively small number of patients, limited follow-up duration, single-centre patient recruitment, and a retrospective design. In addition, the definition of obesity is controversial, and it is unclear whether BMI is the most appropriate measure of obesity. BMI and BSA are crude measures of obesity, and VFA (visceral fat area) or SFA (subcutaneous fat area) may be more appropriate obesity indicators. Computed tomography can be used to accurately assess intra-abdominal fat via measurements of SFA and VFA at the level of the umbilicus [23 **].** However, in clinical practice, SFA and VFA measurements may be difficult and expensive. BMI is a useful, easy, and non-invasive biomarker. Moreover, there is still a controversion among different centres about dosing of chemotherapy for overweight patients due to the concerns of toxicity [36]. In our study, dosage adjustment of chemotherapeutic agents for patients with BSA over 2 m^2^ might have confounded the evaluation of treatment efficacy. However, bevacizumab dosing was applied according to the actual patient weight; thus, patients are supposed to receive ideal planned anti-VEGF therapy.

In conclusion, our results provide the evidence that the BMI of patients prior to initiating VEGF-targeted therapy is a simple prognostic factor for patients with metastatic CRC. Further studies may help determine whether the predictive effects of high BMI are related to either a larger distribution volume of VEGF-targeted therapies, the production of high levels of VEGF by visceral fat, or both. Consequently, patients with high BMIs may not benefit from VEGF-targeted therapy or may require higher dosages. If further validation studies with VEGF-targeted therapies corroborate our results, BMI should be incorporated into clinical patient care and stratification schema for future clinical trials. VEGF-targeted therapies should consider physiological parameters related to the patient in addition to pathologic parameters related to the tumour.
